# Supplementary material for: Structure–function analysis of histone H2B and PCNA ubiquitination dynamics using deubiquitinase-deficient strains
Source: Sci Rep. 2023 Oct 4;13:16731. doi: 10.1038/s41598-023-43969-z (PMC10550974; doi:10.1038/s41598-023-43969-z)

Supplemental Figures For

Structure-function analysis of histone H2B and PCNA ubiquitination dynamics using deubiquitinase-deficient strains.

Kaitlin S. Radmall, Prakash K. Shukla, Andrew M. Leng and Mahesh B. Chandrasekharan

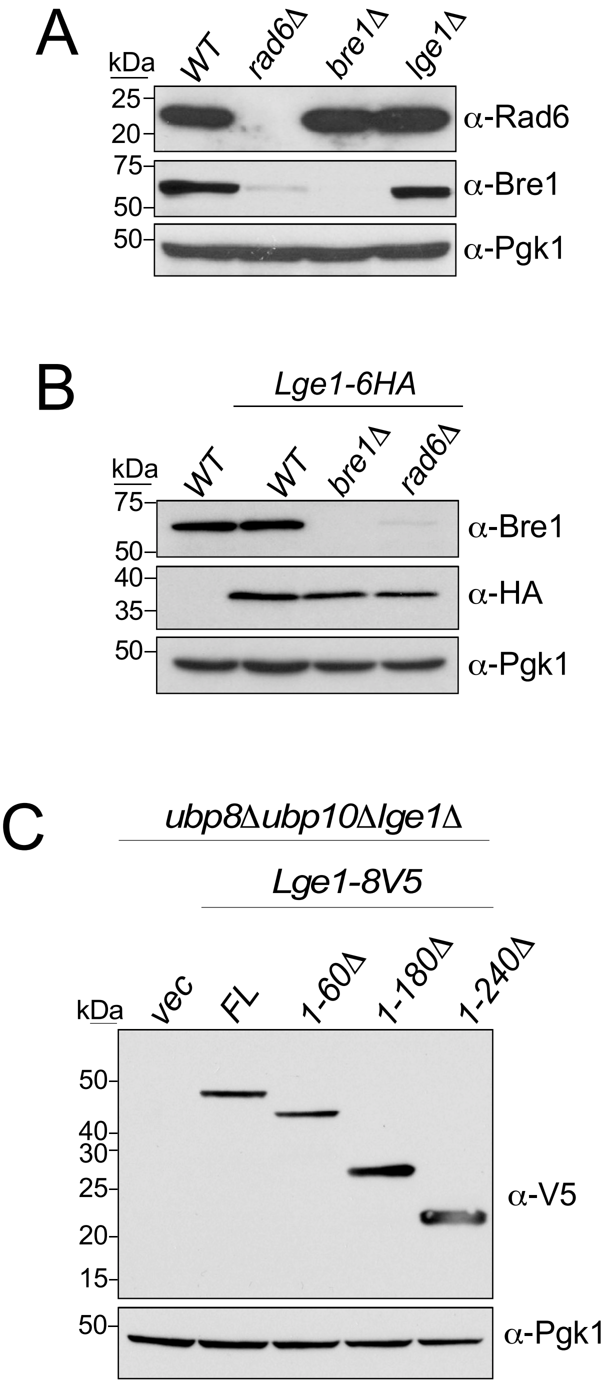

Figure S1. (A) Immunoblots for Rad6 and Bre1 in extracts prepared from WT and indicated null mutant strains. **(B)** Immunoblots for Bre1 and HA epitope-tagged Lge1 in extracts prepared from WT and indicated null mutant strains. Extract from a WT strain without HA-tagged Lge1 served as a negative control. **(C)** Immunoblots for V5 epitope-tagged Lge1 or its N-terminal truncation mutants expressed in *ubp8Δubp10Δlge1Δ* null mutant strain. Extract from a strain transformed with empty vector (vec) served as a negative control. Pgk1 levels served as loading control.

```
P06106|Rad6_Scer MSTPARRRLMRDFKRMKEDAPPGVSASPLPDNVMVWNAMIIGPADTPYEDGTFRLLLEFD 60
P23566|Rhp6_Spom MSTTARRRLMRDFKRMQQDPPAGVSASPVSDNVMLWNAVIIGPADTPFEDGTFKLVLSFD 60
P49459|UBE2A_Hsap MSTPARRRLMRDFKRLQEDPPAGVSGAPSENNIMVWNAVIFGPEGTPFEDGTFKLTIEFT 60
P63146|UBE2B_Hsap MSTPARRRLMRDFKRLQEDPPVGVSGAPSENNIMQWNAVIFGPEGTPFEDGTFKLVIEFS 60
                  *** ***********:::* * ***.:*  :*:* ***:*:** .**:*****:* :.*

P06106|Rad6_Scer EEYPNKPPHVKFLSEMFHPNVYANGEICLDILQNRWTPTYDVASILTSIQSLFNDPNPAS 120
P23566|Rhp6_Spom EQYPNKPPLVKFVSTMFHPNVYANGELCLDILQNRWSPTYDVAAILTSIQSLLNDPNNAS 120
P49459|UBE2A_Hsap EEYPNKPPTVRFVSKMFHPNVYADGSICLDILQNRWSPTYDVSSILTSIQSLLDEPNPNS 120
P63146|UBE2B_Hsap EEYPNKPPTVRFLSKMFHPNVYADGSICLDILQNRWSPTYDVSSILTSIQSLLDEPNPNS 120
                  *:****** *:*:* ********:*.:*********:*****::********:::**  *
                                                149 153
                                                 |   |
P06106|Rad6_Scer PANVEAATLFKDHKSQYVKRVKETVEKSWEDDMDDMDDDDDDDDDDDDDEAD 172
P23566|Rhp6_Spom PANAEAAQLHRENKKEYVRRVRKTVEDSWES--------------------- 151
P49459|UBE2A_Hsap PANSQAAQLYQENKREYEKRVSAIVEQSWRDC-------------------- 152
P63146|UBE2B_Hsap PANSQAAQLYQENKREYEKRVSAIVEQSWNDS-------------------- 152
                  *** :** *.:::* :* :**   **.**..
```

Figure S2. Sequence alignment performed using Clustal Omega for Rad6 and its homologs. Conserved residues (★) and residues with strong (:) or weak similarity (•) are indicated. Uniprot IDs are indicated. Scer, *Saccharomyces cerevisiae*; Spom, *Schizosaccharomyces pombe*; Hsap, *Homo sapiens*.

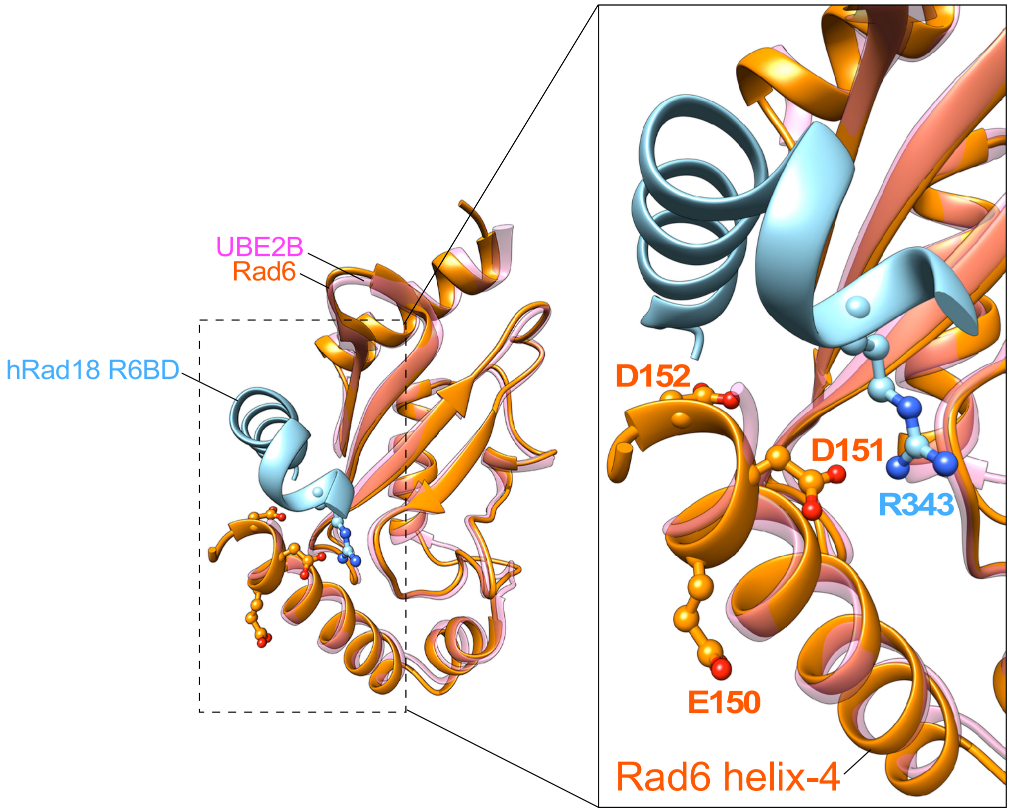

Figure S3. Structure of yeast Rad6 (PDB 1AYZ) superimposed on human Rad6b/UBE2B in complex with Rad18 R6BD (i.e., Rad6 binding domain) (PDB 2YBF). Zoomed image shows the helix-4 terminal negatively charged residues of Rad6 with aspartate 151 of Rad6 in close proximity to arginine 343 of the Rad18 R6BD.

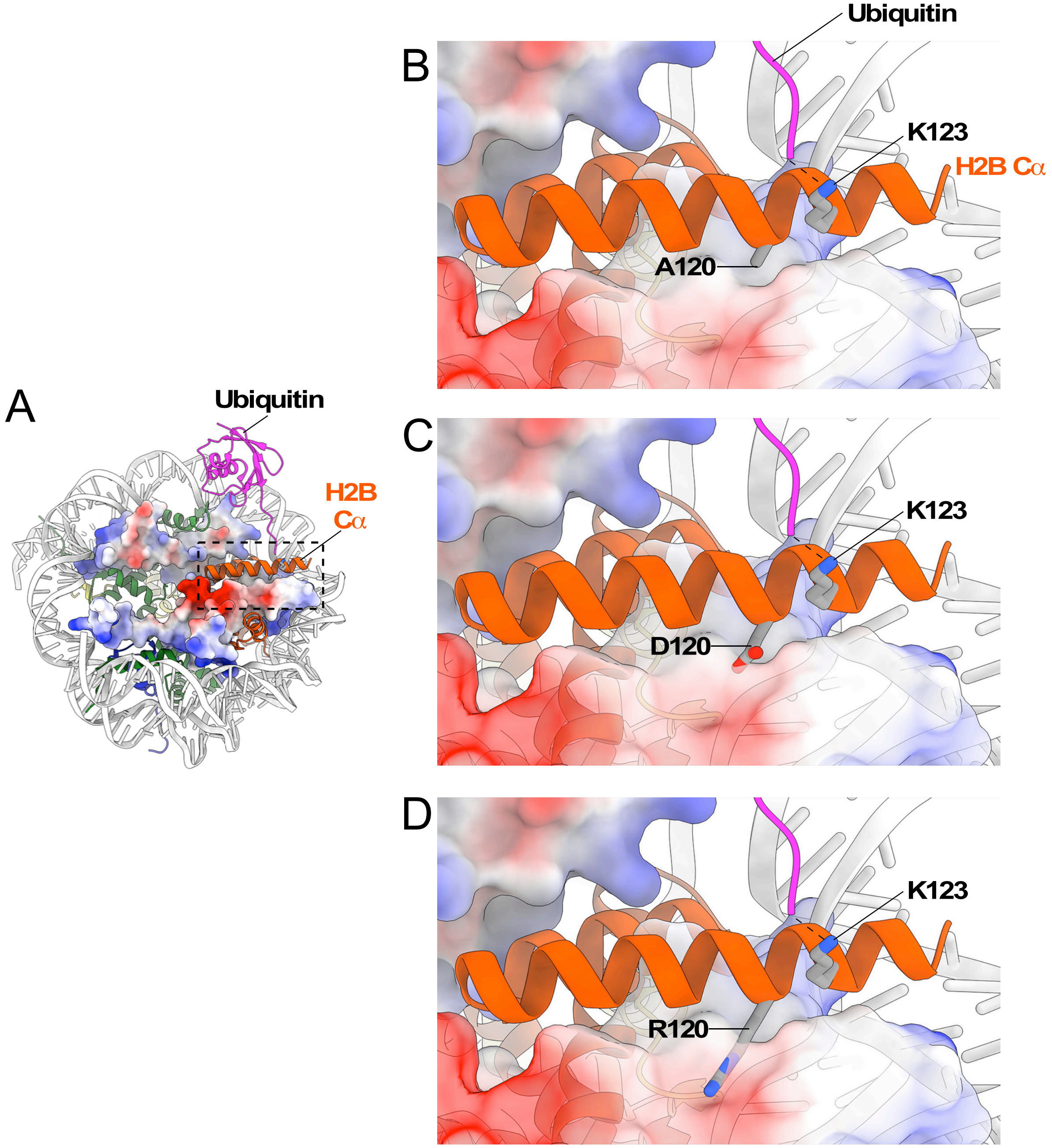

Figure S4. (A) Structure of yeast nucleosome (PDB 1ID3) superimposed on the H2BK120ub1 *Xenopus* nucleosome (PDB 4ZUX). The structure for ubiquitin (pink) was retained to obtain a model for the yeast nucleosome with histone H2B (orange ribbon) monoubiquitinated at K123. Histone H2A and H4 are shown in surface charge representation. Gray, hydrophobic; blue, basic; red, acidic. **(B)** Zoomed image of H2B C-terminal helix (Cα). Histone H2B lysine-123 (K123) and alanine-120 (A120) are shown in ball and stick representation. Dashed line indicates isopeptide bond between K123 and glycine at the C-terminal end of ubiquitin. **(C-D)** Models for nucleosomes with C) aspartate and D) arginine substitutions at position 120 in the H2B C-terminal helix. The negatively charged aspartate (D120) resides in a repulsive acidic or hydrophobic environment likely resulting in a conformational change in the H2B C-terminal helix. On the other hand, the positively charged arginine likely interacts with the neighboring acidic residues to stabilize the helix.

Figure 1

1A

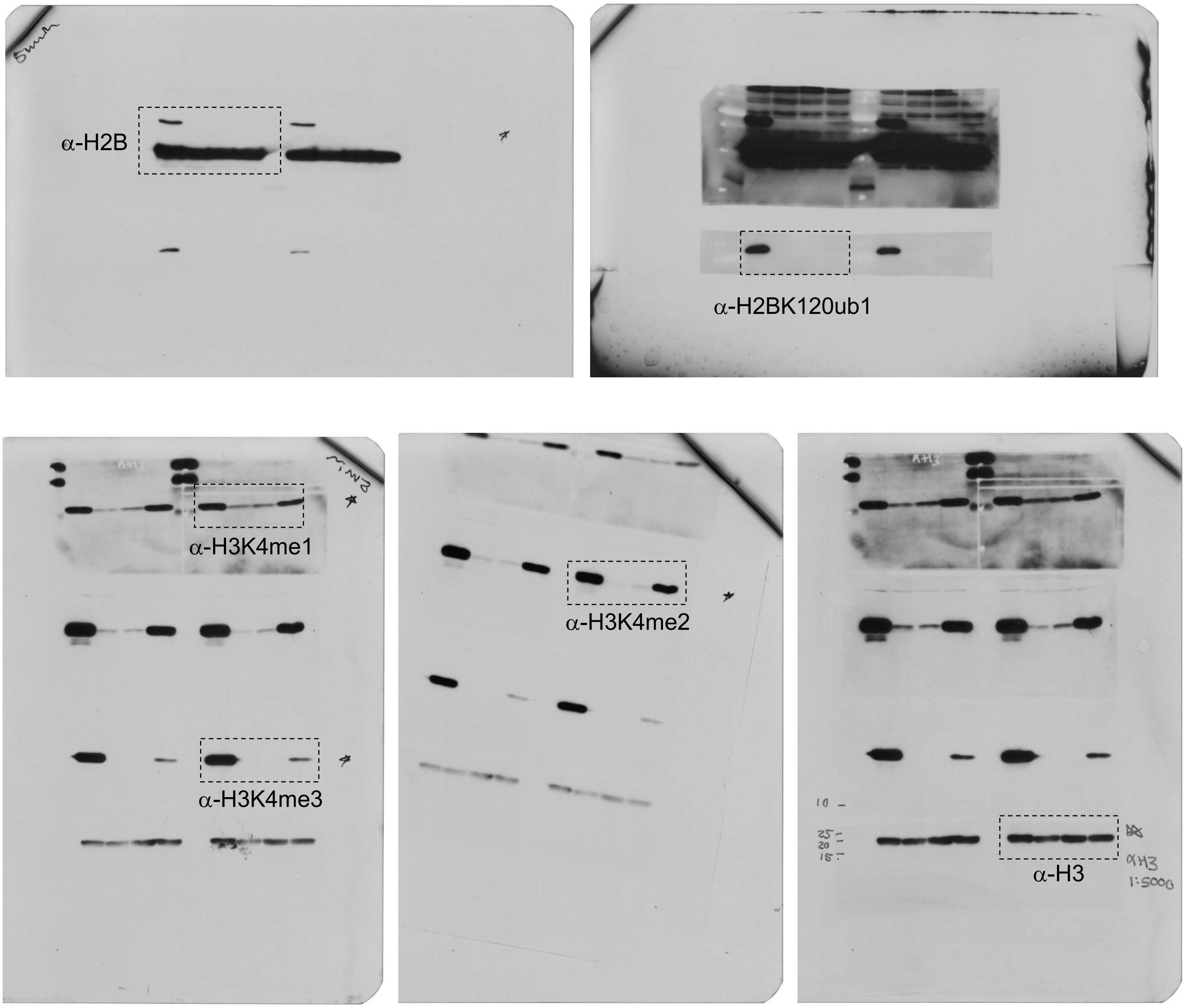

1B

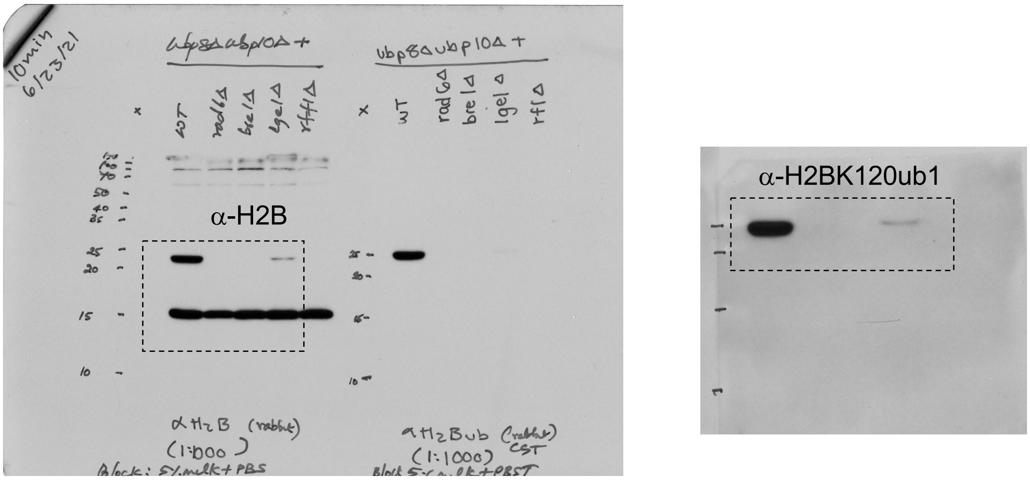

Figure 2

2C

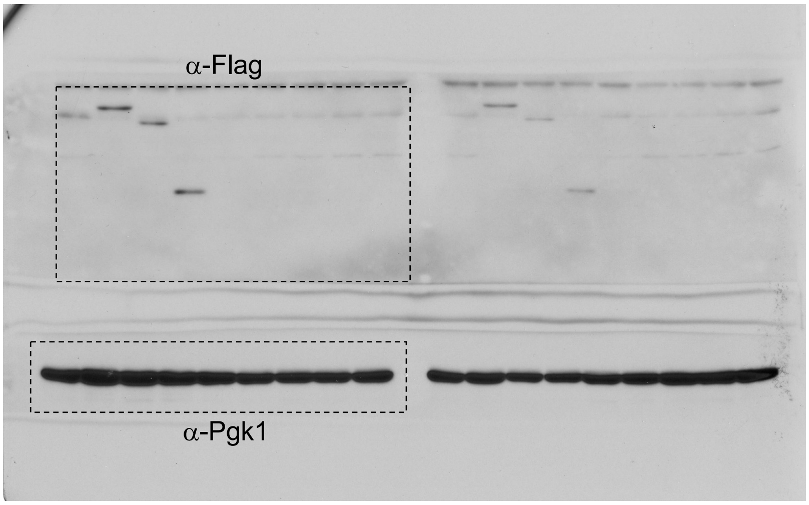

2E

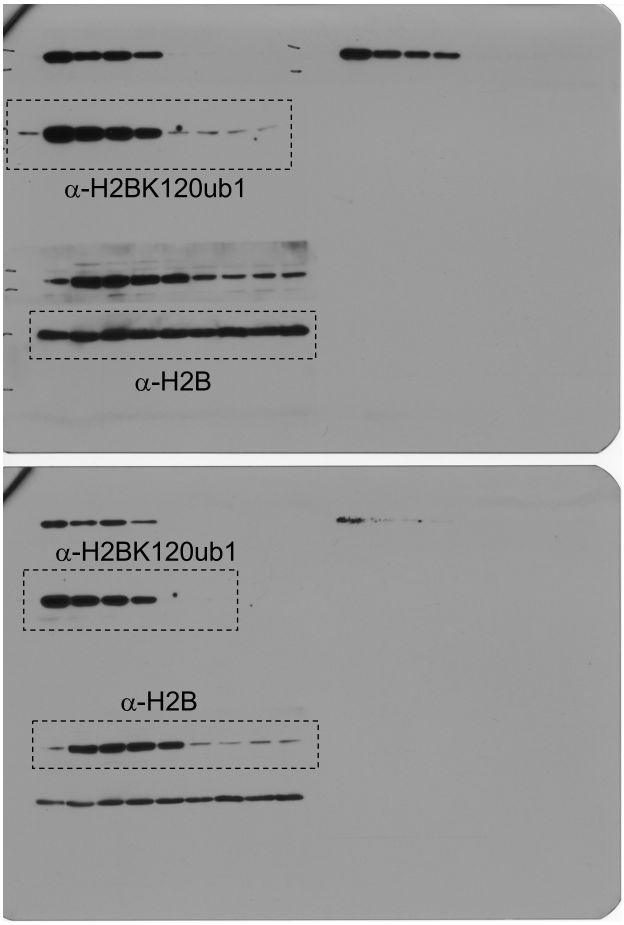

Figure 3

3C

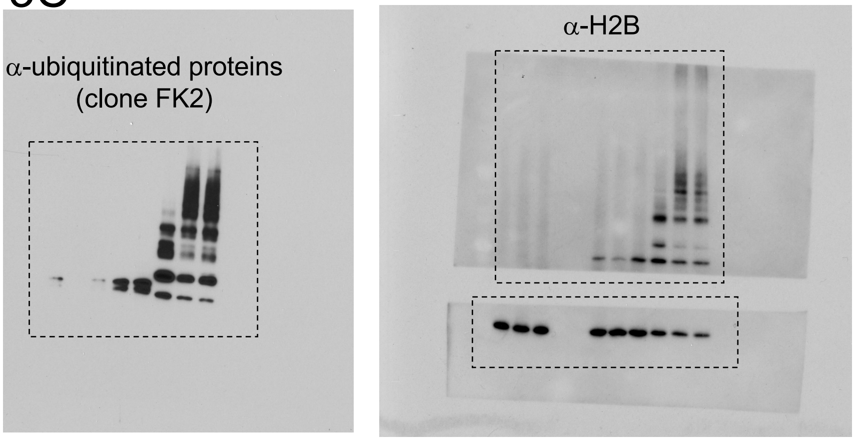

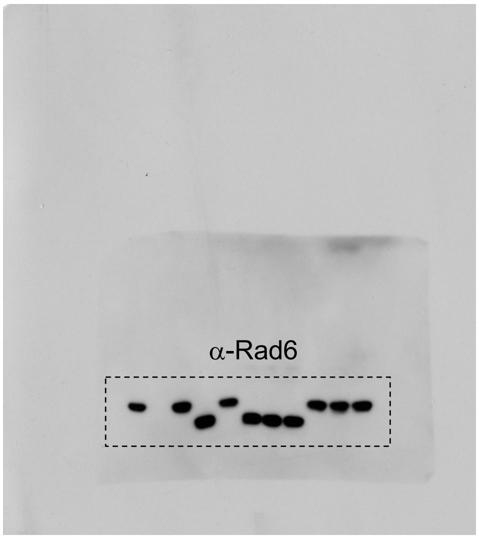

3E

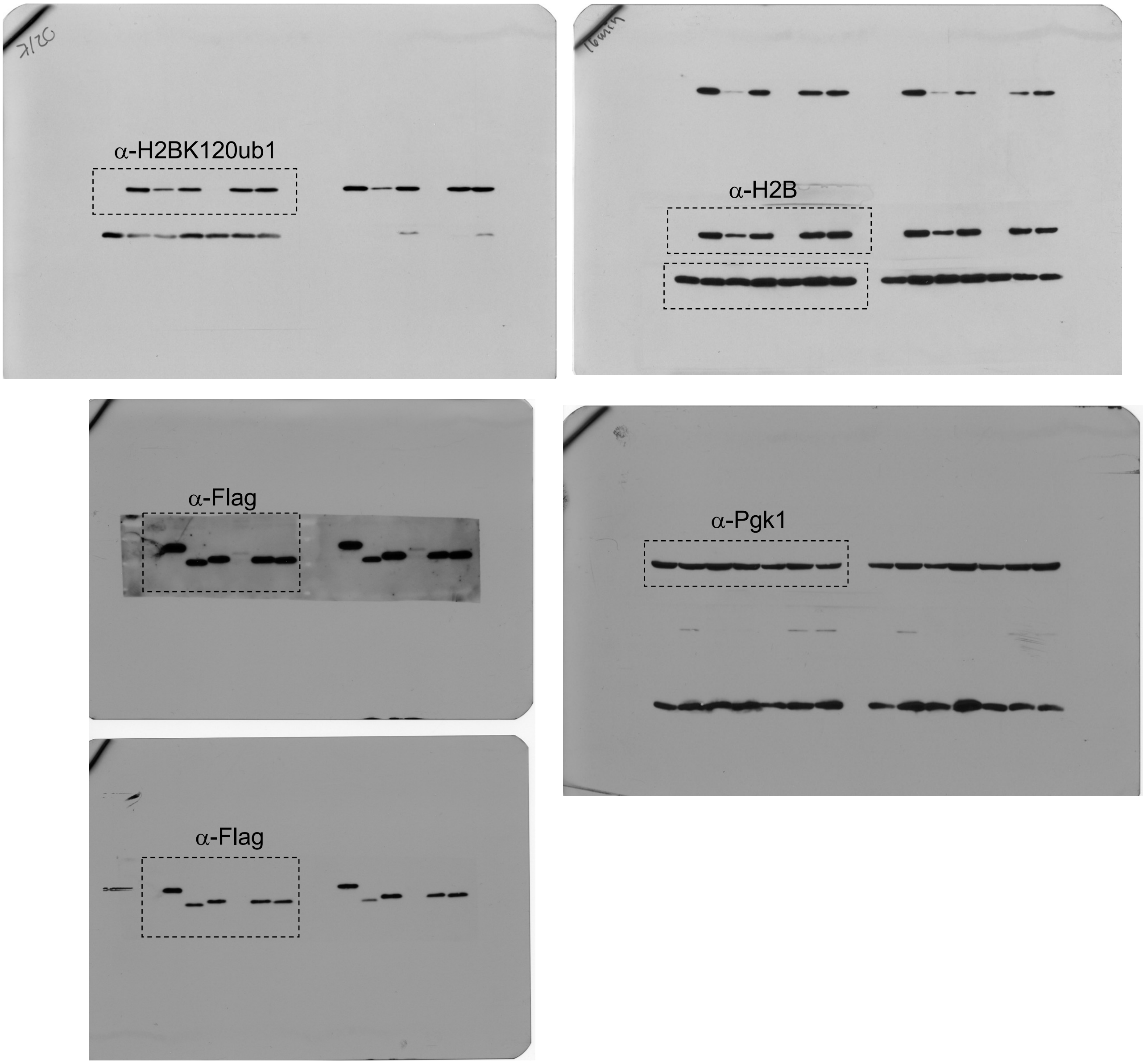

Figure 4

4A

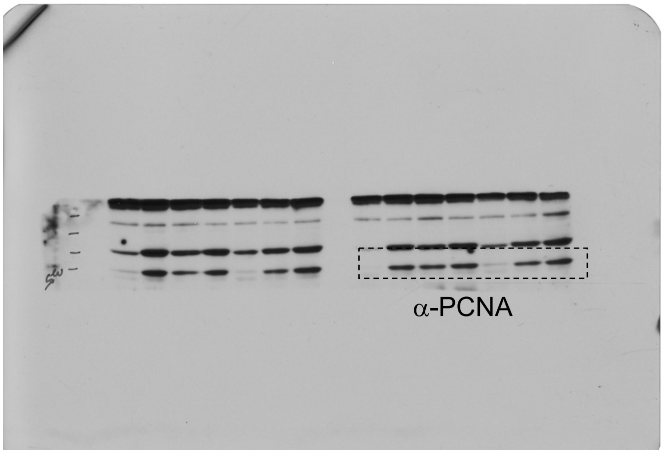
α-PCNA

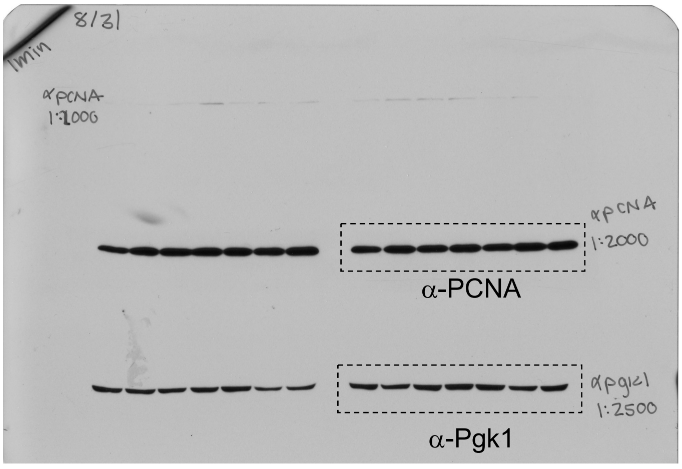
8/31
1min
αPCNA
1:2000
αPCNA
1:2000
α-PCNA
αpgk1
1:2500
α-Pgk1

Figure 5

5C

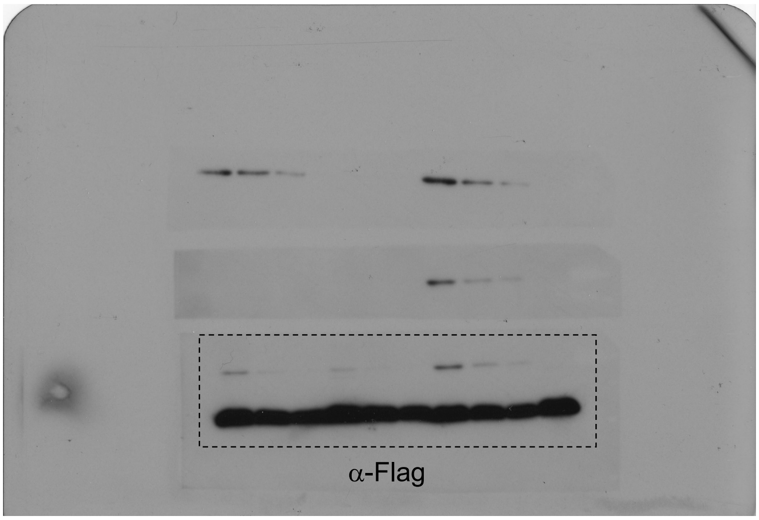

5D

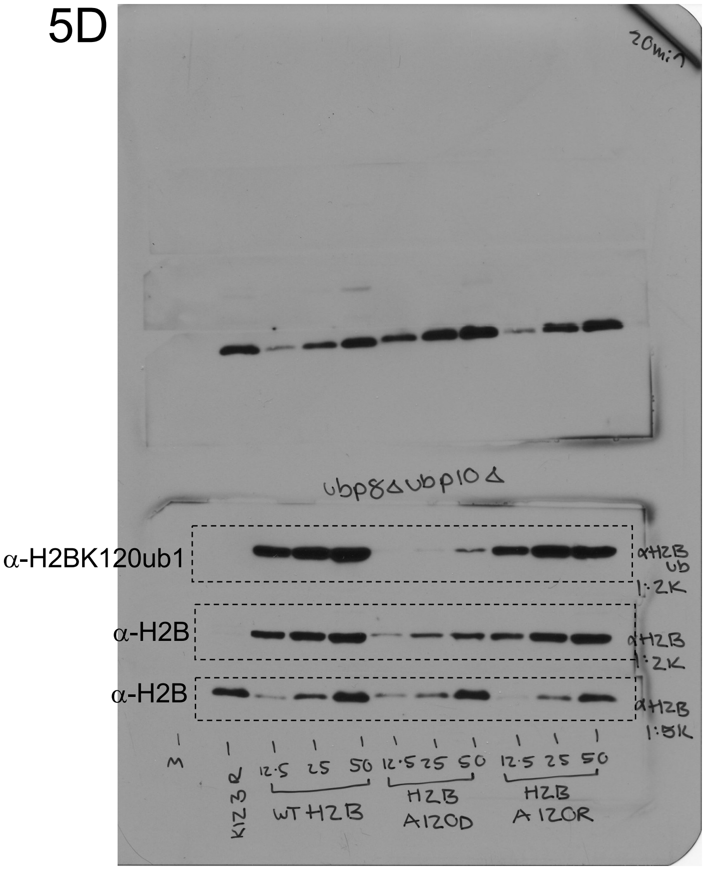

Figure S1

S1A

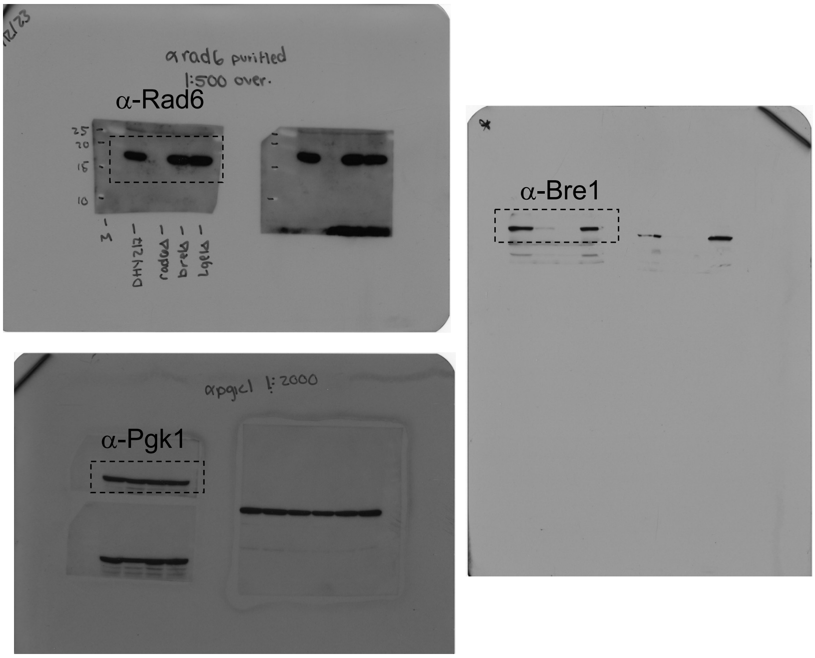

S1B

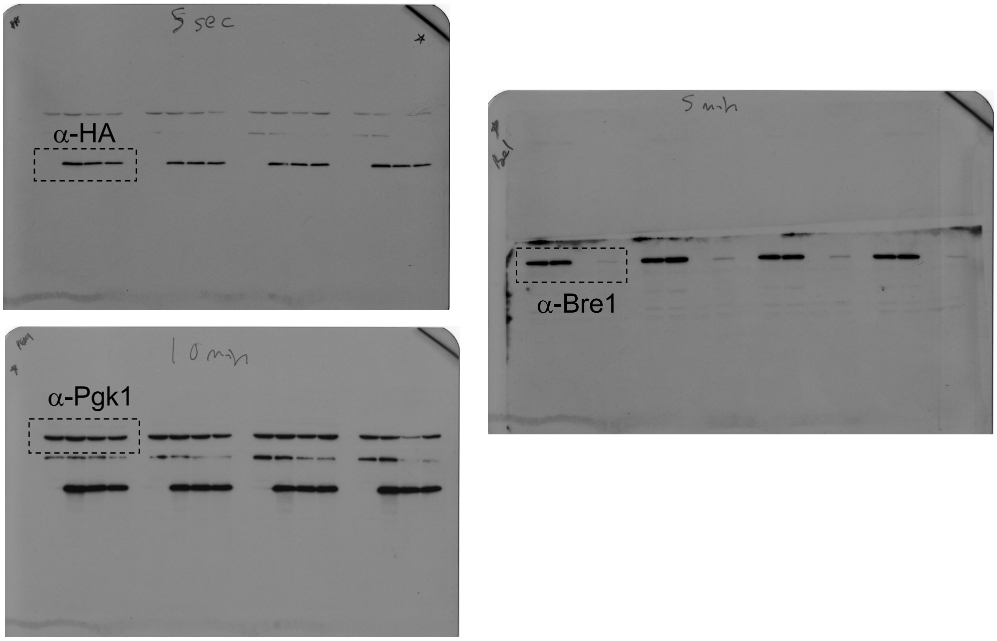

S1C

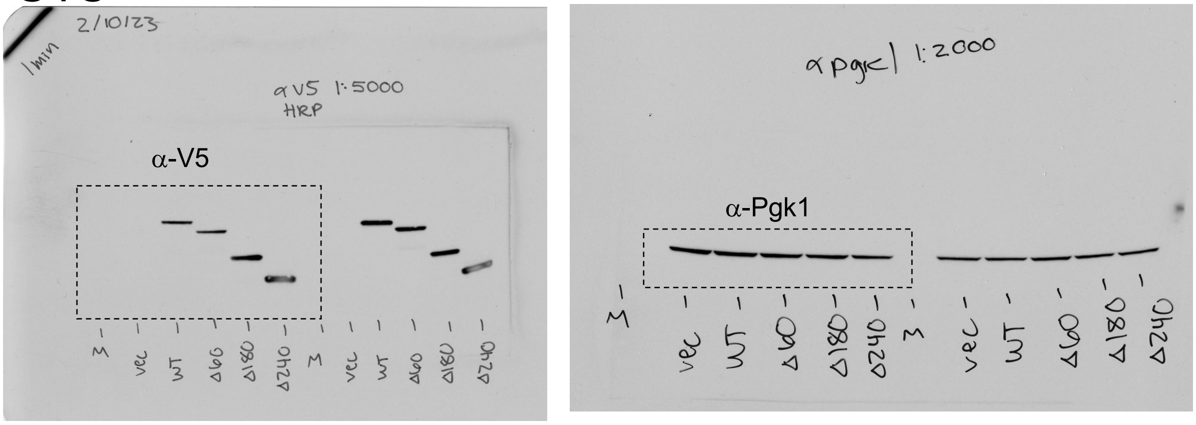

Supplement: Supplementary file 1 — Supplementary Figures. [file 41598_2023_43969_MOESM1_ESM.pdf]
